# Study of the Effect of the Strategy of Heating on the Mudejar Church of Santa Maria in Ateca (Spain) for Preventive Conservation of the Altarpiece Surroundings

**DOI:** 10.3390/s130911407

**Published:** 2013-08-26

**Authors:** Fernando-Juan García-Diego, Ángel Fernández-Navajas, Pedro Beltrán, Paloma Merello

**Affiliations:** 1 Departamento de Física Aplicada. (U.D. Industriales), Universitat Politècnica de València, Av. de los Naranjos s/n, Valencia 46022, Spain; E-Mails: afnavajas@fis.upv.es (A.F.N.); pbeltran@fis.upv.es (P.B.); 2 Centro de Tecnologías Físicas, Unidad Asociada ICMM- CSIC/UPV., Universitat Politècnica de València, Av. de los Naranjos s/n., Valencia 46022, Spain; 3 Instituto Valenciano de Conservación y Restauración de Bienes Culturales (IVC+R), Complejo Socio-Educativo de Penyeta Roja s/n., Castellón 12080, Spain; E-Mail: palomamerello@outlook.com

**Keywords:** preventive conservation, Spanish cultural heritage, temperature and humidity sensors, climate control system

## Abstract

The mudéjar church of Santa María (Ateca) is valuable for its architecture and the altarpiece contained inside. Ateca is a village with continental climate characterized by cold winters and hot summers. In this paper we are interested in analysing the effect of temperature and relative humidity (RH) changes produced by the heating system on the altarpiece. Therefore, a monitoring system of 15 temperature and 15 relative humidity sensors was installed with a recording frequency of a data point per minute. The main contribution of this paper is the quantitative study of the effect of the heating system on the thermo-hygrometric parameters using statistical techniques such as ANOVA, mean daily trajectories or bivariate plots, and the proposal of an innovative dynamic contour plot. As results, the heating system produces a substantial increase (decrease) of temperature (RH) causing an hourly variation of these physical parameters detrimental to the conservation of the altarpiece, especially in its higher areas.

## 1. Introduction

In last decades an increasing interest has developed in all areas of conservation, with an interdisciplinary approach. Preventive conservation, as a method of work, aims to control the deterioration of the artwork before it occurs. Currently, preventive conservation measures are acknowledged as important for safeguarding cultural heritage (CH), both in terms of preserving CH and also reducing the cost of future conservation measures [1].

It is recognized that works of art in unheated churches often remain in relatively good condition over centuries, while rapid signs of degradation are observed after heating has been introduced [2]. The indoor climates of unheated buildings are essentially governed by the outside climate modified by the building envelope. The standards [3,4] recommend that, for the best preservation of materials which are sensitive to moisture, the relative humidity (RH) ranges should replicate the long-term local climate and that the RH fluctuations centred on this local RH level must be kept to a minimum. Heating can introduce serious destabilization to the natural indoor climate in a church.

In particular, wood is a material that undergoes noticeable changes in response to variations in the physical parameters (RH and temperature) characterizing the environment in which it is placed. The main mechanical properties of wood are its elastic and strength properties. In general, many mechanical properties are affected by changes in moisture content below the fibre saturation point [5]. At constant moisture content and below approximately 150 °C [5], mechanical properties are approximately linearly related to temperature.

The wood supports of paintings are complex structures in themselves [6], which may experience uneven moisture changes, and consequently uneven dimensional responses, on opposite faces of a panel owing to a lower permeability of the painted face to the flow of moisture [7]. Dimensional change is perhaps the most important consequence of moisture interaction with wood affecting any artwork. Wood shrinks as it loses moisture and swells when it gains moisture. Therefore, these contractions and dilations hinder predicting the dimensional change of a given wooden object accurately and the empirical formulae given in wood handbooks can merely be used to estimate the changes [8].

In recent decades an increasing interest in knowing how RH and temperature can modify the mechanical properties of wood producing deterioration in artworks where this is the main material or base has been developed. Some papers have studied this concept from an experimental approach [9–12]. In [12], numerical modelling was used to follow the evolution of the moisture content gradient and the stress field resulting from the restrained differential dimensional response across a wooden cylinder, simulating sculptures. However, these studies relate to equilibrium conditions which do not usually coincide with the dynamically changing environments to which historic wooden objects are often exposed. For this reason, in later works [13–15] historic wooden objects have been monitored *in situ*. In relation to artworks exposed to heating systems inside churches, some studies performed in churches in Norway have helped to establish that the need for conservation is due to the fact that the climate in heated churches in cold climates is unfavourable for painted wooden objects [16,17].

In particular, recent works in the literature [2,18,19] analyse the influence of RH and temperature on the state of conservation of altarpieces located in churches. In [18], triangulation laser displacement sensors were applied to the continuous *in situ* monitoring of the response of wooden altarpiece in the church of Santa Maria Maddalena in Rocca Pietore, Italy, to fluctuations in temperature and relative humidity caused by the use of a heating system. In another paper [2], the evolution of cracks in some figures of the altarpiece was monitored according to RH variations, and the main conclusion obtained was that the variation in the moisture content caused dimensional changes in the wood; the response, however, was characterized by ranges and rates which varied considerably with the thickness of the wooden elements. The authors of [19] employed Digital Speckle Pattern Interferometry (DSPI) and Speckle Decorrelation (DIC) to perform condition surveys of a wooden altarpiece in the church of Hedalen, Norway. The authors of [19] only consider an easy sampling method to be used as an alternative to visual inspection of the piece under study by conservators, but they do not perform an exhaustive monitoring of the physical parameters that are influencing the deterioration.

None of the aforementioned studies analyse in depth and quantitatively the behaviour of the temperature and RH with a sensors mesh that considers different heights and orientations in the altarpiece. One of the main contributions of this work is to consider the effect of the microclimate on the different parts of the altarpiece.

Ateca is a village in the province of Zaragoza in the Autonomous Community of Aragon (Spain). Located at the southwest of the province, at the confluence of the rivers Jalón and Manubles, Ateca is at 606 m above sea level (Latitude: 41°19′51″ N, Longitude: 1°47′36″ O) [20].

**Figure 1.**
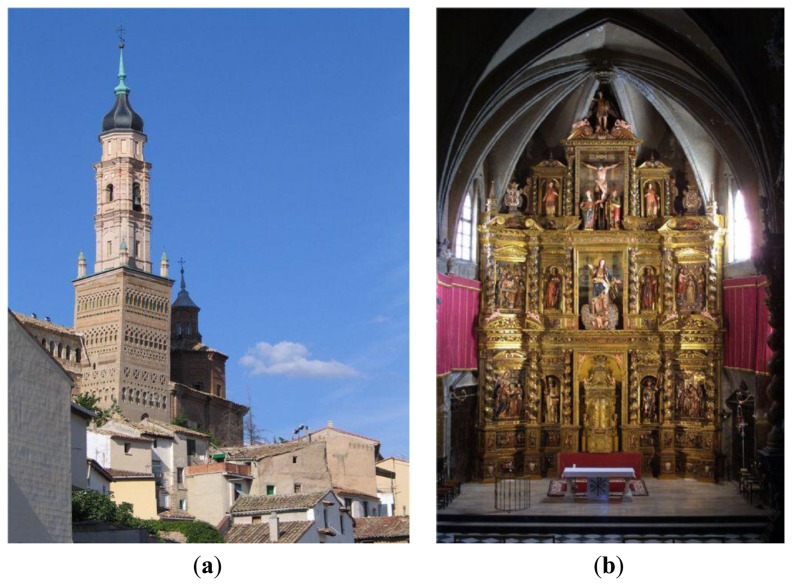
(**a**) Exterior Photograph of Mudejar Church of Santa María in Ateca. (**b**) Altarpiece. Photographs from the Prensa Diaria Aragonesa SA archive taken by Santiago Cabello.

In the village centre there stands the Mudejar parish church of Santa María. Mudejar is a style of Iberian architecture and decoration, particularly in Aragon and Castile, dating to the 12th to 16th centuries and strongly influenced by Moorish taste. In contrast to other churches of the same period, Mudejar churches are mainly made of brick and plaster, instead of ashlar stone [21]. Santa María of Ateca is a temple of a single nave, a seven-sided polygonal apse and chapels between the buttresses. It has a square tower (Figure 1a) and two sections, minaret structure with stairs covered by barrel vaults and simple ribbing. The tower was built during the second half of the thirteenth century while the Mudejar church was constructed in the fourteenth century.

The altarpiece (Figure 1b) develops the most important events in the life of the Virgin Mary, and was made between 1650 and 1657. The author of the relief was Martin of Almunia, from Ateca. Bernardo Ibañes was the author of the sculptures, and they were polychromed by Juan Lobera and his sons Jusepe and Francisco Lobera [22].

Ateca features an extreme climate, with a mean daily RH between 91.97% and 11.94% and a mean daily temperature between 38.48 °C and −5.59 °C. Therefore, a single heating strategy is adopted. The church is heated rapidly, shortly before and during services, to a more comfortable temperature [23,24] with a hot air heating system through the floor of the church.

The vault had repainting and detachment problems. Thus, in 2011, a restoration work performed by the company Albarium S.L. began. Taking advantage of the installed scaffolding a monitoring system was installed on the altarpiece to control both the dome and altarpiece, which has major conservation problems.

It was decided to implement a monitoring system for recording a full year of data of the physical parameters involved in conservation with more influence on wood, *i.e.*, temperature and humidity, both in the front and the back of the altarpiece.

This paper aims to document the conservation conditions, the possible causes of deterioration and the effect of the heating system on the microclimatic conditions affecting the altarpiece of the church of Santa Maria in Ateca (Spain), to prolong its preservation as much as possible (with corrective measures if necessary), being the first time that a microclimatic study is performed in a Mudejar church. For this purpose, the recorded data are analysed by descriptive univariate and multivariate statistical methods [25,26] recently employed in cultural heritage, serving this example as a working methodology for similar cases.

## 2. Materials and Methods

### 2.1. Monitoring System

A total of 15 probes were installed, 14 in the interior of the temple distributed in the front and the back of the altarpiece, and an additional probe placed on the sill of a window as an outdoor climate control (Table 1). All probes contain an 8-pin small-outline integrated circuit (SOIC), model DS2438 (Maxim Integrated Products, Inc., Sunnyvale, CA, USA) that incorporates a direct-to-digital temperature sensor with an accuracy of ±2 °C as well as an analogue-to-digital voltage converter which measures the output voltage of a humidity sensor (HIH-4000, Honeywell International, Inc., Minneapolis, MN, USA). Because each DS2438 contains a unique silicon serial number, multiple DS2438s can exist on the same data bus. This allows multiple sensors that can be used in the system simultaneously with only one data line (1-wire communication protocol). The HIH-4000 RH sensors were calibrated in the laboratory with a saturated solution of salt as explained in [27].

As specified by the manufacturer, the voltage output of the HIH-4000 sensors is proportional to voltage supply thus the exact value of the voltage supply was measured for each RH sensor once all probes and connections were installed and the calibration curves of each sensor were corrected.

Three electric wires come out from each probe: one wire for +5 V DC power supply, one for ground and another for data transfer. The measurements were recorded in digital format by a microcontroller, to which all sensors were connected in parallel. Recorded data were downloaded monthly to a pen drive.At the time the monitoring system was installed, it was decided to study whether there were differences between the different areas of the altarpiece (front *vs.* back, and height). However, due to technical difficulties during installation, sensors could not be perfectly distributed for a complete statistical sampling; therefore most sensors of the front were located at the top of the altarpiece (Table 1).

### 2.2. Data

The sensors were installed on 10/14/2011. As the monitoring period for analysis we considered those data taken from 10/15/2011 to 09/04/2012, however some data had errors, so a total of 80 days were eliminated, which means a total of 245 available days. The outdoors sensor (#15) started measuring from 01/04/2012 to 09/04/2012.

The sensors were installed with a data acquisition frequency of 60 data points per hour (1 data point every minute). Thus, each sensor is capable of recording 43,200 data points per month (30 days × 24 h/day × 60 data points/h). Therefore, in this study we have a data matrix of 352,800 (245 days × 24 h × 60 min) rows and 15 columns (sensors) each one.

The software used for the storage and management of recorded data [28] allows detecting and eliminating those anomalous data caused by a punctual error in the sensor data collection or a numerical register that does not fit within the physically possible minute increments of that parameter. In this case, a minute variation of ±5 °C and ±5% of RH is considered for removing data.

### 2.3 Statistical Analyses

Different exploratory and comparative statistical analyses are used in this work. In order to study if the differences among different positions of the sensors were statistically significant, a multifactor ANOVA was carried out considering different factors as appropriate. We worked with the following factors: day, height (0–5 m (low), 5–10 m (intermediate), 10–13 m (high)), orientation (B/F), season (10/14/2011–3/31/2012 “cold”, 4/1/2012–5/9/2012 “warm”), and the climate control system (ON/OFF). Different statistical parameters were calculated for each hour and each temperature and RH sensor: the average and the hourly variation.

In order to study the effect of height and orientation where the probe was located, different ANOVA models were tested with these parameters. ANOVAs were performed using the software Statgraphics 5.1 [29].

Mean trajectories allows an easy identification of deviations with respect to the target trajectory when cycles are clearly marked. Mean trajectories is a plot commonly applied in the control of batch chemical processes, however this kind of plot is not frequently used in cultural heritage microclimate monitoring studies [26]. The time series of temperature or RH recorded by one data-logger reflects the parameter evolution along time. Temperature and RH mean daily trajectories were plotted in order to discuss the dissimilarities among probes and to identify abnormal patterns.

We also analyse contour plots, as done in [30]. The graduation of the parameter yields by triangulation from the physical parameter value in two points connected by a straight line (in this case a sensor and the closest one). After doing this for all sensors, points with equal graduation are connected with splines, obtaining a contour plot of the physical parameter. The dynamic contour plots have been similarly made, but considering as points in the plane the hours of the day and the months of the year.

Finally, bivariate plots have proven to be a simple technique that can be interpreted by visual inspection and giving similar results to cluster analysis [26]. A bivariate plot was obtained to compare the difference between the average value of temperature before switching the heating system and the maximum temperature during it is switched on *versus* (T MAX-T AVG) the difference between the average value of RH before switching the heating system and the minimum RH during it is switched on (RH AVG-RH MIN).

## 3. Results and Discussion

### 3.1. Characterization of the Outdoor and Church Microclimate

**Figure 2.**
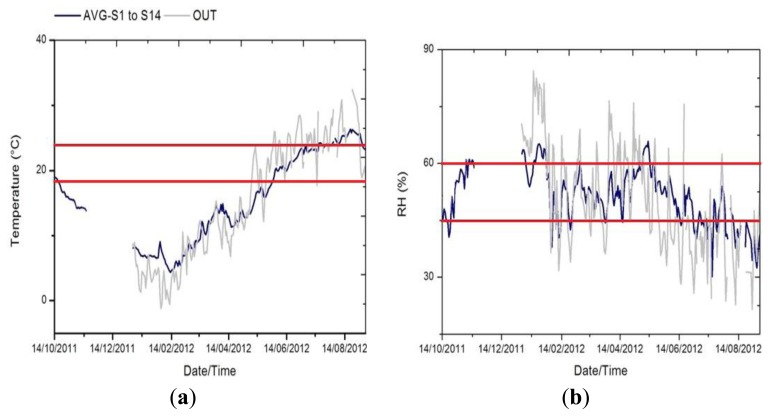
Time series of the average of inner sensors (from #1 to #14) (blue) and the outdoors (whose data begin on 01/04/2012, gray), (**a**) temperature, (**b**) RH.

In Figure 2 we see how the temple dampens the external variability, and also appreciate the extreme climate in Ateca, reaching negative temperatures in winter and above 30 °C in summer. Average trajectories of both parameters (RH and temperature) were inspected for the identification of inappropriate conditions that might be harmful for the wood altarpiece according to the standard UNI and DM 10/2001 [3,4], which recommendation for carved wood is 19–24 °C for temperature (maximum daily variation of 1.5 °C) and 45%–60% RH (maximum daily variation of 2%).

For the monitoring period, approximately 80% of the points (32% for RH) of the hourly average temperature (RH) of the altarpiece exceed the recommended values (Figure 2).

### 3.2. Microclimatic Characterization of the Altarpiece

As seen in the previous section, temperature exceeds more often than RH the recommended limits, reaching values predominantly lower than the recommended because winter temperatures in the temple reach values below 19 °C, while in the case of RH the limits are exceeded mostly in summer. There exist differences between seasons and next we characterize the altarpiece discussing the main differences between the orientation of the sensors for the different seasons.

**Figure 3.**
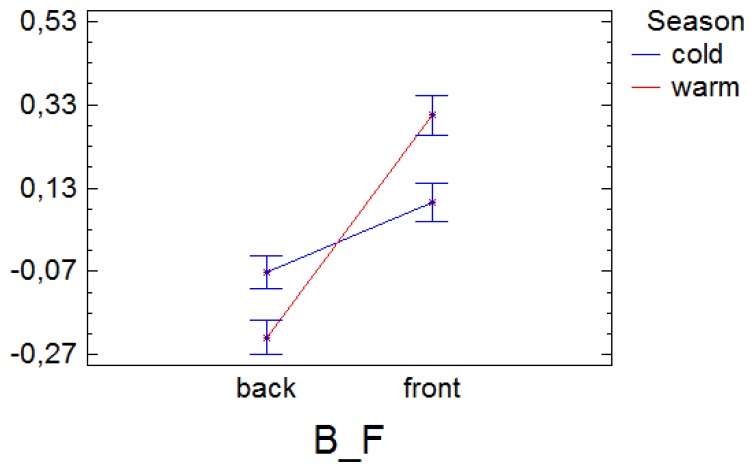
Interaction plot of the factors orientation (B/F) and season (cold = blue, warm = red), for the ANOVA analysis of the differences to the average temperature (residuals of an ANOVA of the temperature and the factor season).

In Figure 3 we analyse the interaction between the sensor orientation and the season. In this ANOVA, the factor season results not meaningful (*p* value > 0.05) because we are working with their residuals to see differences to the average of that season, *i.e.*, the values of the vertical axis are the differences to the average for that season. However, the interaction is significant.

For temperature, the difference to the average is greater in the front than in the back of the altarpiece, however that difference results even more marked in summer as a result of the higher variability (variance) in the data during this season.

**Figure 4.**
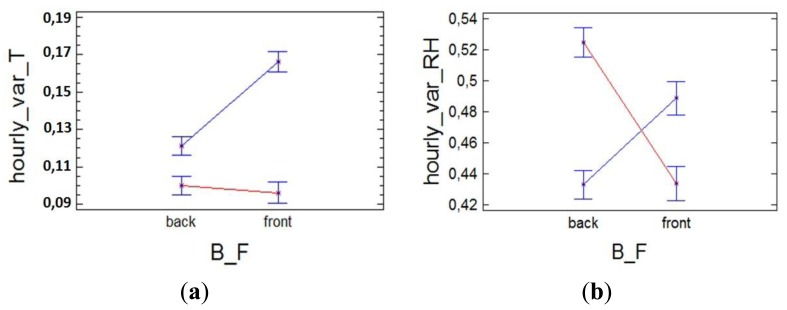
Interaction plot of the factors orientation (B/F) and season (cold = blue, warm = red), for the ANOVA analysis of the hourly variation of (**a**) temperature, (**b**) RH.

However, we think it is possible that the differences between the front and back (B/F) can be given in terms of the hourly variability of temperature. Thus, the ANOVA (Figure 4) indicates that in the case of temperature there are only significant differences between B/F in the cold season, which could indicate that these differences in hourly variation are caused in part by the switching of the heating system.

In the case of RH, in summer, when there is no heating, and most RH variability occurs in the back (Figure 4b). However, in winter, the highest variability occurs in the front, which could indicate that this greater variability is induced by the climate control system on the same way that seemed to indicate the temperature. Note that although these differences are small, lower than the sensor error, due to the fact an average of a large amount of data is calculated, these differences are significant.

### 3.3. Analysis of the Effect of The Climate Control System

It is required to determine those specific or regular events that cause an increase (decrease) of temperature (RH) due to the switching of the climate control system, and, after studying the mean daily trajectories of the average of all inner sensors for each day of the week, the following events have been detected:

- Saturdays at 20:00, coinciding with the main religious service of the week. These peaks are explained by the switching on of the heating system at 19:00 to heat the temple for the Mass; the heating system shuts off between 20:30 to 20:45, at the end of the religious service. The heating system is switched on from 2/4/2012 to 5/5/2012. From November 2011 to January 2012 Masses were held at another local church due to restoration works.
- Saint Blas festivity (2/1/2012–2/3/2012).
- From 3/26/2012 to 3/31/2012, coinciding with the preparations for the Easter processions.

ANOVAs were conducted considering the climate control system factor, which will take the value “ON” for those cases when it is switched on as a result of an event. In this way we want to analyse if the heating system produces significant changes in temperature and RH.

**Figure 5.**
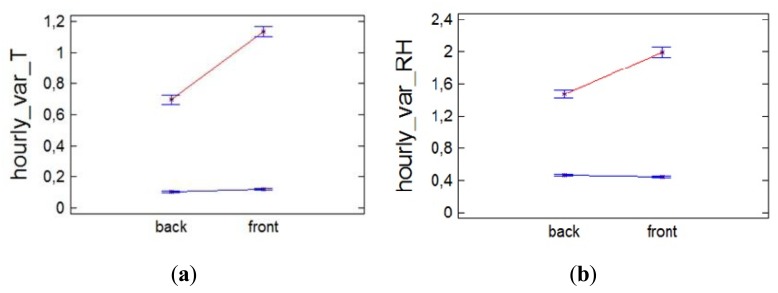
Interaction plot of the factor climate control system (blue = OFF, red = ON) and orientation (B/F), for the ANOVA analysis of the hourly variation of (**a**) temperature, (**b**) RH.

**Figure 6.**
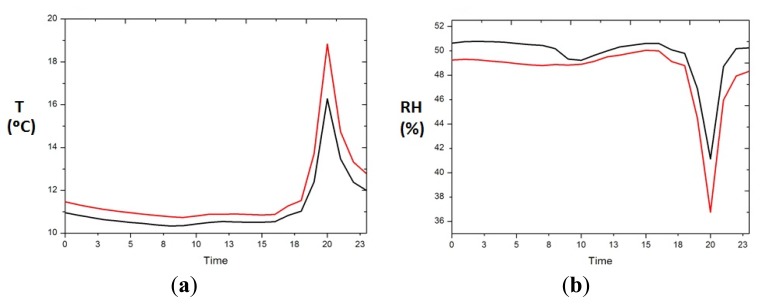
Daily mean trajectories for back (black) and front (red), for days when the heating system is switched on, (**a**) temperature, (**b**) RH.

ANOVA indicated that significant differences exist between the front and the back, and especially for those situations when the heating system is switched on (Figure 5), so next we analyse the mean daily trajectory of the front and back sensors for days when the heating system is switched on (Figure 6). The sensors of the front have all day a temperature approximately higher 0.5 °C (1.5% lower RH) than those in the back, but when the heating system is switched on the difference between front and back is accentuated reaching 3 °C of temperature (4% RH).

A contour plot is performed to visually assess those areas that are most affected by the heating system.

**Figure 7.**
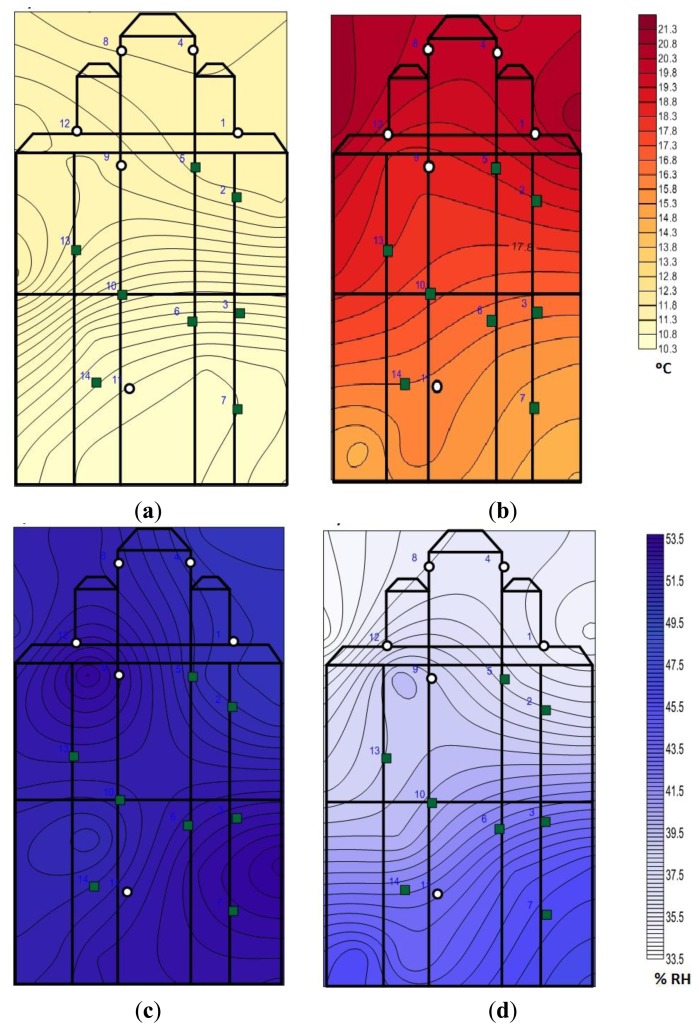
Contour plots for days when the heating system is switched on, (**a**) for the average temperature before switching (2 h), (**b**) maximum temperature during the system is switched on, (**c**) for the average RH before switching (2 h), and (**d**) minimum RH during the system is switched on. Circles represent sensors on the back of the altarpiece and squares represent those sensors located in the front of it.

Notice that temperature and RH are practically constant in the entire altarpiece when the heating system is not working (Figure 7a,c). However, for the hours when the heating system is switched on there is a vertical gradient of temperature and RH (Figure 7b,d), but with these studies it is unknown if the differences are significant (both in temperature and in RH), so we perform ANOVA analysis considering the height (low, intermediate and high) as a factor.

**Figure 8.**
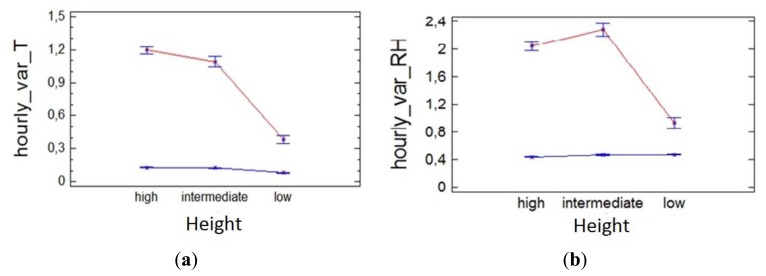
Interaction plot of the factors climate control system (blue = OFF, red = ON) and height, for the ANOVA analysis of the hourly variation of (**a**) temperature, (**b**) RH.

The bigger hourly variation clearly occurs as consequence of the heating system and especially in the upper and middle areas of the altarpiece as hot air rises (Figure 8). Note that the hourly variation of temperature and RH exceeds the maximum recommended daily variation for the standards, so considering that these recommended values are for daily variations we can assert that an hourly variation of this magnitude will be very detrimental to the conservation of the altarpiece.

Since most of the sensors in the front are placed at the top, the B/F effect is possibly masked with the predominant effect of the height when the heating system is switched on (Figure 7b,d). The question arises whether the significant difference between back and front sensors are due to its orientation or rather due to the height at which they are located.

Note that in Figure 4, the hourly variation differences between B/F are not significant for temperature in the warm season (when the heating system does not work). In the case of RH, these differences are significant but with opposite sign (in the warm season there is more variation in the back, and in the cold season in the front).

**Figure 9.**
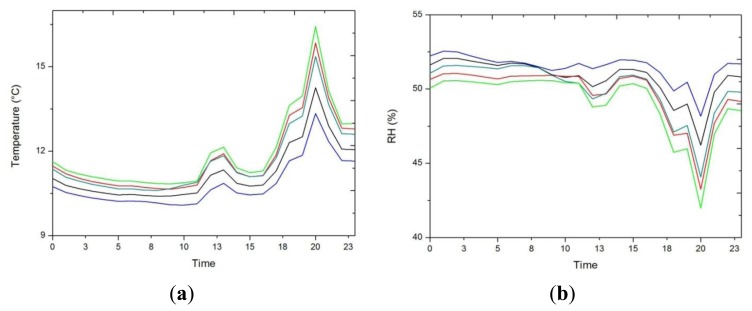
Mean daily trajectories, for days when the heating system is switched on, of the average of sensors at the back (black), the average of sensors at the front (red), the average of sensors at low height (blue), the average of sensors at intermediate height (green) and the average of sensors at high height (lime). (**a**) Temperature. (**b**) RH. Note the similarity between the red and lime trajectories, although softened the red one since it also includes sensors #9 and #11.

This suggests that the differences attributed to the factor B/F when the heating system is switched on (Figure 5) would actually be a consequence of the fact that the sensors in the front are mostly from the top area (note the similarity in shape between red and lime trajectories in Figure 9). Thus, when the heating system is working the main differences can be found between heights, masking this factor the difference between B/F, as can be seen in Figure 7b,d where the gradient is mainly vertical. It would be interesting to investigate the interaction of factor B/F and height, but there is no suitable experiments design because there are no all the possible combinations of heights and positions due to technical difficulties during installation.

**Figure 10.**
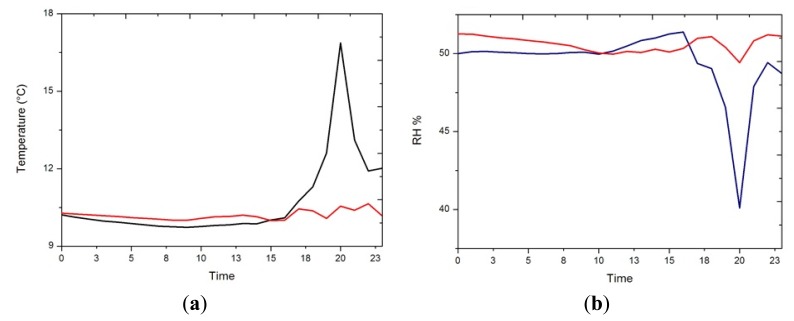
Daily mean trajectories of sensors #1, #4, #8, #12, for Saturday (black) and Monday (red), from 2/1/2012 to 5/5/2012 (period when heating strategies are followed), (**a**) temperature, (**b**) RH.

After performing ANOVA analysis with height factor, we found that the heating system mainly affects the sensors at the upper area (sensors #1, #4, #8 and #12). Thus, the normal behaviour of these sensors on days when no religious events take place (Mondays) *versus* days when the heating system is switched on (Saturdays) are compared. In Figure 10 we can observe how when the heating system is switched on an average increase of 7 °C of temperature (and a decrease of 11% RH) occurs in the range of 1 h.

After verifying that there are significant differences in height as a result of the heating system and evaluate these differences by quantitative (ANOVA) and qualitative (mean daily trajectories and contour plots) methods, we propose to evaluate a bivariate plot to check if they achieve similar results and propose these techniques as an alternative methodology, easier to interpret, for use primarily for conservators who are non-scientists.

**Figure 11.**
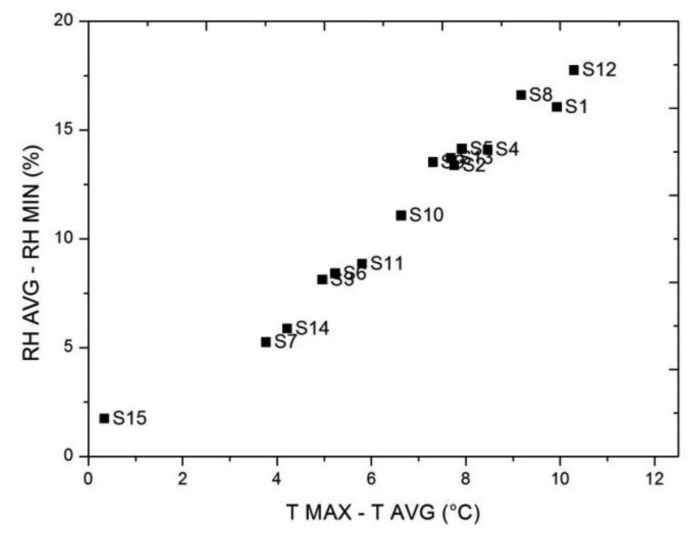
Bivariate plot of temperature (horizontal axis) *versus* RH (vertical axis), of the difference between the average before the heating system is switched on (2 h) and the maximum (minimum for RH) when it is working (from 2/1/2012 to 5/5/2012).

In Figure 11 we see that the main differences between sensors caused by the heating system are produced according to height. It also appears that there might be some difference, although lower, between front and back as sensor #11, located at similar height to #14 and #7, but in the front, reflects a bigger temperature increase (2 °C) and a bigger RH decrease (3%). This fact is not clearly reflected in the contour plots (Figure 7) highlighting another benefit of using bivariate plots. However, we note that the interaction between the height and orientation (B/F) has not been empirically verified by ANOVA due to complications during installation of the sensors, but possibly there exist significant differences between B/F when the heating system is working.

**Figure 12.**
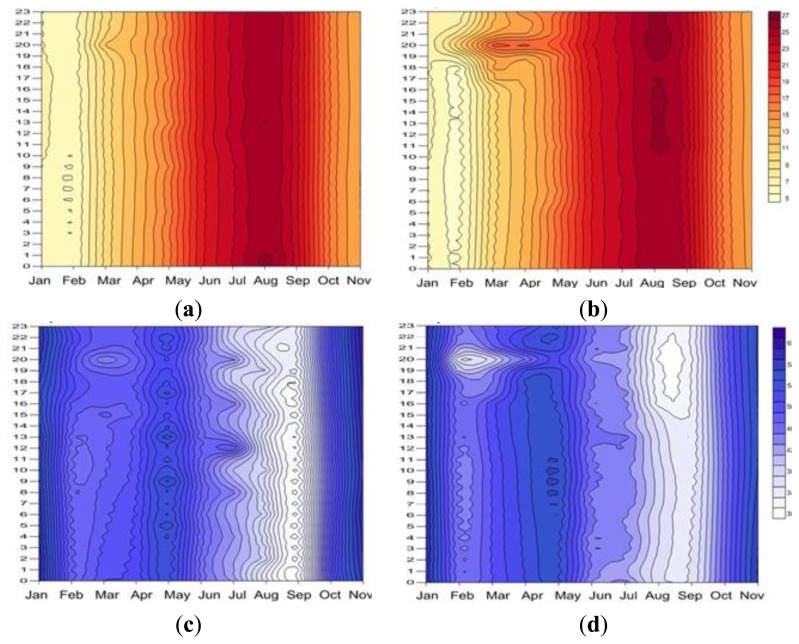
Annual contour plot of mean daily trajectories, of the average of sensors #1, #4, #8 and #12. The horizontal axis represents the months of the year (from January to November) and the vertical axis the hours of the day (0–23). (**a**) Temperature on Mondays, (**b**) temperature on Saturdays, (**c**) RH on Mondays, (**d**) RH Saturdays.

Then an alternative plot is proposed (Figure 12), for simple visual inspection, to evaluate the differences in mean daily trajectories produced by the heating system (in this case comparing days when a religious event takes place and days when no event takes place) and its dynamic behaviour over the year. In our case, since we already know the sensors which are further noting the changes produced by the heating system (#1, #4, #8 and #12) we perform this plot for the average temperature of these sensors. However, note that this graph can also be used with the average of all sensors or, for comparative purposes, for one or more sensors separately.

Figure 12 allows us to visually compare the annual change experienced by the mean daily trajectories. Thus, for a traditionally uneventful day (Figure 12a,c) it is observed how temperature remains constant during the day with the annual gradation typical of seasonality of temperature, which is reflected in the plot by almost parallel vertical lines. Similar results are obtained for RH. However, for Saturdays (Figure 12b,d) of these months when the heating system is working (from February to April) an increase of temperature between 19 and 21 h is produced coinciding with the celebration of Mass in the temple (reflected in the chart by “islands” of different tone). We also see that temperature seems to indicate that the first Saturday of September a Mass or concentration of people occurs in the temple. Studying this fact we realize that it coincides with the festivity of the Virgin of the Peana (from 6th to 10th September), being the 8th of September Saturday, (Figure 12b,d) the festivity of the Virgin.

This proposed plot allows an easy inspection of a dynamic problem as the temperature variation along the day, also considering its typical variation along the year.

## 4. Conclusions

The techniques used have been able to quantify the effects of the heating system on temperature and RH, parameters that determine the conservation of the altarpiece. These techniques allowed us to determine when the heating effects are more pronounced and their hourly evolution.

A quantitative methodology is proposed, based on ANOVA analysis and a graph of simple interpretation, based on contour plots, that combines the power of mean daily trajectories and the dynamic nature of the time series of temperature and RH.

A proper experimental design is important, however not always is possible so take into account the deficiencies of the design and employ techniques to save them is of great interest.

The “on” switch of the heating system has been detected at different moments due to the celebration of Masses and religious events resulting in a rapid hourly increase of the temperature at 7 °C and lowering of 11% of RH in the sensors located in the highest area (#1, #4, #8 and #12).

On the other hand, the gas heater emission of CO_2_ and H_2_O (which would raise the RH) is not affecting that physical parameter, since the figures show when the climate control system is working RH decreases as a result of a temperature increase.

## Figures and Tables

**Table 1.** Position in which the sensors were installed, specifying height (meters) and orientation (Back = B, Front = F).

**Sensor (S)**	**Heigth (H)**	**Back (B)/Front (F)**	**S**	**H**	**B/F**	**S**	**H**	**B/F**	**S**	**H**	**B/F**
			
1	10.2	F	2	8.3	B	3	4.7	B	4	12.6	F
5	9	B	6	4.3	B	7	2.9	B	8	12.6	F
9	9	F	10	6	B	11	3.2	F	12	10.5	F
13	7.23	B	14	3.2	B	15	OUTDOOR		-	-	-
